# 3D-bioprinted all-inclusive bioanalytical platforms for cell studies

**DOI:** 10.1038/s41598-020-71452-6

**Published:** 2020-09-04

**Authors:** Roya Mazrouei, Vanessa Velasco, Rahim Esfandyarpour

**Affiliations:** 1 Medical School, Stanford University, Palo Alto, CA USA; 2 Department of Electrical Engineering, University of California, Irvine, CA USA; 3 Department of Biomedical Engineering, University of California Irvine, Irvine, CA USA; 4 Henry Samueli School of Engineering, University of California, Irvine, CA 92697 USA

**Keywords:** Nanobiotechnology, Biomedical engineering, Techniques and instrumentation, Biotechnology, Cancer, Engineering, Nanoscience and technology

## Abstract

Innovative drug screening platforms should improve the discovery of novel and personalized cancer treatment. Common models such as animals and 2D cell cultures lack the proper recapitulation of organ structure and environment. Thus, a new generation of platforms must consist of cell models that accurately mimic the cells’ microenvironment, along with flexibly prototyped cell handling structures that represent the human environment. Here, we adapted the 3D-bioprinting technology to develop multiple all-inclusive high throughputs and customized organ-on-a-chip-like platforms along with printed 3D-cell structures. Such platforms are potentially capable of performing 3D cell model analysis and cell-therapeutic response studies. We illustrated spherical and rectangular geometries of bio-printed 3D human colon cancer cell constructs. We also demonstrated the utility of directly 3D-bioprinting and rapidly prototyping of PDMS-based microfluidic cell handling arrays in different geometries. Besides, we successfully monitored the post-viability of the 3D-cell constructs for seven days. Furthermore, to mimic the human environment more closely, we integrated a 3D-bioprinted perfused drug screening microfluidics platform. Platform’s channels subject cell constructs to physiological fluid flow, while its concave well array hold and perfused 3D-cell constructs. The bio-applicability of PDMS-based arrays was also demonstrated by performing cancer cell-therapeutic response studies.

## Introduction

Traditionally, biomedical research has relied on animal models or two-dimensional (2D) cell cultures. Animal models, though one of the most commonly used systematic models, provide a limited understanding of human-specific biology of different tissues. This is due to several reasons, such as fundamental differences between humans and animals^1^, low throughput studies, in addition to the ethical concerns^2^. Animal testing is not cost-effective, considering the cost to provide care, food, and shelter for the animals. Moreover, it is still possible that animal models can show promising results for drug treatments that can be harmful when tested on human subjects^3^. On the other hand, despite the broad applications of 2D-cell cultures, these models only interact with their microenvironment in two dimensions, which in most cases cannot properly represent physiological conditions^4^. Several studies have shown that 2D-cell constructs possessed altered cell polarity, mechanical cues, biochemical signals, and cell–cell interactions^5^. Recently, there has been an emergence of three-dimensional (3D) cell models that better capture the complex cellular microenvironment than the conventional 2D models. 3D-models have shown improvement and relevance in vivo cell structure and function, where features such as the cell type, cell morphology, cell propagation, as well as, differentiation are more precisely represented^6–13^. Furthermore, one should note that each year, billions of dollars are wasted because of preclinical 2D cell culture failure in predicting drug safety and efficacy in humans, which also slows the development of treatments for patients in need. There are many examples in the literature regarding the substantial differences in drug response between cells cultured in 2D vs. 3D formats^14–16^. Numerous studies have shown that cell responses to drugs in 3D-culture are improved from those in 2D in terms of modeling functionality of in vivo tissue, which illustrates the benefits of using 3D-based models for preclinical drug screens. There is enough evidence that 3D-cell structure models may be preferable for drug screening applications^17–19^. In fact, in a 3D-environment, cells grow naturally, which affects the way cells interact with each other and their micro-environment. Therefore, when drug candidates are being tested using cell-based assays, the methods of cell culture utilized should imitate the most natural and possible in vivo environment. Thus, 3D-cell culture is the most natural tissue-mimicking method for drug discovery. In recent years several principles and technologies such as 3D-tissue and organ culture are merged with 3D-printing and microfluidic approaches to address the issues regarding the proper representation of cell models and to capture complex human physiology in vitro. A combination of these emerging technologies enables the development of unique platforms to control perfusion and allow for quantitative pharmacology models, which usually are referred to as "organ-on-a-chip" (OOC) devices. OOCs, in brief, incorporate several approaches with two main aims of employing perfusion to mimic the physiology and microenvironment of cells and employing (3D) cultures of different cell types. Cell types in OOCs represent a desirable subset of organ or tissue functions such as metabolism or excretion, useful in studying quantitative systems pharmacology models. OOC systems can range from single organ systems to more complicated multi-organ systems in a single circulation^20–22^. These particular devices have the potential to enter the pharmaceutical industry as a rapid and cost-effective drug screening platform. However, these devices have been hindered due to manufacturing challenges, such as the requirements of sophisticated and expensive fabrication systems^23,24^. One critical consideration is that OOC manufacturing demands flexibility and a broad resolution range in order to achieve the physiological relevance of the organ of interest. Conventional fabrication techniques such as^25,26^ lithography and laser micro-machining are limited by the necessity of complicated, expensive facilities, and equipment^27^. Low-cost methods such as laser cutting and molding have been used as well but are hindered by low resolution^24,28^. On the other hand, 3D- printing innovation has revolutionized traditional fabrication and manufacturing technology, especially for micro-scale structures and devices in the past decade. Moreover, 3D-printers usually have higher repeatable performance compared to other rapid prototyping technologies such as soft lithography and infrared laser micro-machining^29^. Using 3D-printing technology enables researchers with limited production abilities and industry experts to execute fast prototyping of designs that are complicated to manufacture with conventional processes such as machining or casting. Additionally, the development of biocompatible systems for 3D-printing has been especially promising for tissue engineering applications. 3D-cell models are usually derived from several conventional methods, including extracellular matrix scaffold, spinning bioreactors, and air–liquid interface methods^28^. Recently, 3D-bioprinting technologies have been implemented to derive and improve 3D-cell culture models^30^. Unlike conventional methods, 3D-bioprinters enable automated production of fast, reproducible, and customized 3D-cell structures. In 3D-bioprinter based models, features such as the size, shape, volume, instructive biomolecules, and cell content are better defined, uniquely designed, and controlled^31^. Perhaps the most valuable advantage of 3D-bioprinters is the ability to deposit scaffold, polymer materials, and cells simultaneously or consecutively. The capability and flexibility of generating complex and diverse geometries that represent human physiology with the same tool used to disperse cell structures streamline the process of producing cell analysis platforms. The printing automation eliminates the need for skilled personnel and allows for mass production arrays of both the platform and cell models. Also, the demand for in vitro 3D-organ models has increased to achieve a better prediction of the human body responses to new candidate drugs. Nonetheless, as mentioned above, using animal models for drug testing is not always practical, and ethical issues regarding animal use have been a topic for debate in the past years. An alternative option is 3D-bioprinted 3D-models, which have the potential to mimic cell viability, metabolic activity, and the vital reactions of organs or tissues.

## Results and discussion

### Design principles and considerations

The ability of OOCs systems to mimic cell functions that is representative of organ-level physiology allows for the generation of novel platforms to examine disease mechanisms, progressions, and testing of candidate treatments^20,21^. However, the design, production, and successful applicability of OOC devices require several critical deliberations including rapid manufacturability of fluidic perfusion systems, development of cell models with sufficient physiological microenvironment relevance, and combined execution of these two into a single cohesive platform. For in vitro drug screening testing systems, for instance, it is critical to examine different therapeutics or their concentrations on the same model population. The incorporation of fluidic handling devices such as micromixers enables the preparation and delivery of different doses of (or combinations of) therapeutics within a chip flow circulation. Development, characterization, and validation of such devices are some of the efforts in this study.

On the other hand, the generation of suitable 3D-bioprinted constructs requires the optimization of multiple parameters simultaneously to achieve the desired resolution for diverse 3D-constructs. 3D-bioprinting involves the precise layering of cells, biologic scaffolds, and growth factors capable of creating 3D-cell constructs for a variety of applications^27^. The use of 3D-bioprinting requires optimization of parameters such as extrusion pressure, printing speed, and the use of proper nozzles to achieve desired resolutions, which is part of our reported study, as explained in “3D-bioprinting for fluidic handling systems” section. Moreover, the generation of accurate representations of the complicated cell physiological microenvironments, similar to those found in 3D-cell models, is extremely important. This is an especially crucial feature for drug testing platforms to provide 3D-cell models, as it is shown that these models can improve preclinical drug efficacy or failure predictions^27^. Such 3D-cell models must be able to replicate the intricate cell environment while the platform must encompass arrayed features (for high throughput testing) that can be flexibly designed to various sizes, and compartments to promote the desired environment. The use of 3D-bioprinting technologies lends itself to the flexible printing of both the platforms and 3D-cell structures. In this work, considering these requirements, we adapted 3D-bioprinting technology to develop customized OOC-like platforms along with 3D-cell structures for the analysis of 3D-cell culture models as well as the study of cell-therapeutic responses. Materials also play a critical role in 3D-bioprinting since the material properties can affect the fabrication process and the application of the 3D-printed components. Scientists developed several materials for these applications according to their characteristics such as transparency, printability, viscosity, and flexibility^32,33^. Transparency of matrices is one of the important features in fluidic handling devices (e.g., microfluidics) since visual observations are usually required in such devices. In biological applications, the biocompatibility of these materials is another critical parameter. Many researchers extensively utilized a variety of polymers and thermoplastic materials for 3D-bioprinting applications. Solidifiable fluids such as photopolymer resins, temperature-sensitive polymers, and ion cross-linkable hydrogels are the most commonly used materials for 3D-bioprinting in recent years^25,34–36^. Polydimethylsiloxane (PDMS), for instance, is one of the most commonly used materials to manufacture microfluidic devices because of its excellent transparency and biocompatibility^29,37^. Here, we first demonstrate 3D-bioprinted spherical and rectangular cell constructs within 3D-bioprinted well arrays. One should note that the physically defined well arrays can potentially be useful for growing cells onto the scaffold with the ultimate goal of in vivo transplantation to the bone, cartilage, ligament, skin, vascular, neural, and skeletal muscle tissues^38^. It is also worth noting that the recent studies confirm that cells cultured using well arrays exhibit different enzyme expression levels and drug reactivity compared to culturing in traditional 2D format^39^. Besides material selection, the use of 3D-bioprinting requires other critical considerations, including limiting induced mechanical stress during 3D-bioprinting of the structures, supplying cells with nutrients during post-printing culture, using suitable bioinks, and monitoring and maintaining printed constructs viability and proliferation process. For instance, extrusion pressures and nozzle sizes should be optimized to not inflict shear stress on cells suspended in viscous fluids and decrease the cells’ survival rates. In this work, we aimed to carefully consider and optimize these requirements, as explained in “3D-bioprintability and viability monitoring of 3D-HCT116-constructs” section. In this study, human colorectal cancer cells were used, since colorectal cancer is the third most prominent cause of cancer-related deaths among women and men^40^. Though many developments in early detection and treatment have been shown for colon cancer, there is still room for therapeutic improvement, especially for those patients with invasive and aggressive tumors presenting drug resistance^41^. One of the other main considerations in 3D-bioprinting of cell constructs is maintaining the post-viability of printed cells. Here, we printed and monitored arrays of 3D-bioprinted encapsulated cell constructs, with both spherical and rectangular shape, within 3D-printed PDMS well arrays. The spherical model was selected as they resemble the compact arrangement of tumors and the rectangle shape was selected as they resemble a simple model of a vasculature structure. Experiments showed successful cell viability, as explained in the “Viability analysis of 3D-HCT116-constructs” sub-section. Lastly, to more closely mimic the in vivo environment by replicating the mechanical cues such as fluid flow (i.e., shear stress) that tissues are subject to within the body, we continued the effort by the development of an OOC-like platform. This device is a perfused well-based fluidic handling platform, consists of two main parts: fluidic delivery channels and concave wells. The fluidic delivery channels were designed to subject cell constructs to physiological fluid flow, and at the same time, deliver nutrients. The platform also consisted of a concave well array that held the 3D-human colon cancer cell constructs (HCT116). Next, we aimed to optimize the process to maintain shape and stability at physiological temperature, while at the same time simulating an optimal microenvironment (e.g., adhesion sites) for cells. For this, we used, Gelatin methacryloyl (GelMA), a gelatin-based bioink that can maintain shape and stability at physiological temperature, has proven biocompatibility, and provides mammalian cells with a milieu that resembles some essential properties of their native environment. GelMA also has high content of arginine-glycine-aspartic acid (RGD) useful for cell attachment, and target sequences of matrix metalloproteinase (MMP) that assist in cell proliferation which are desirable attributes necessary for our printed cell structures^42–44^. GelMA HCT 116 cell constructs were printed into the well arrays and perfused with media using the fluidic handling channels. In this platform, printed GelMA HCT 116 cell structures formed ring/toroidal shapes within a day after printing, which are useful structures to model the tubular geometry of the colon, where tumors are usually found attached to the inner wall of the large intestine. Finally, preliminary drug screening investigations were performed with 2D HCT116 cell models within 3D printed PDMS well arrays.

### 3D-bioprinting for fluidic handling systems

#### Characterization of rapid manufacturing of fluidic handling systems

**Figure 1:**
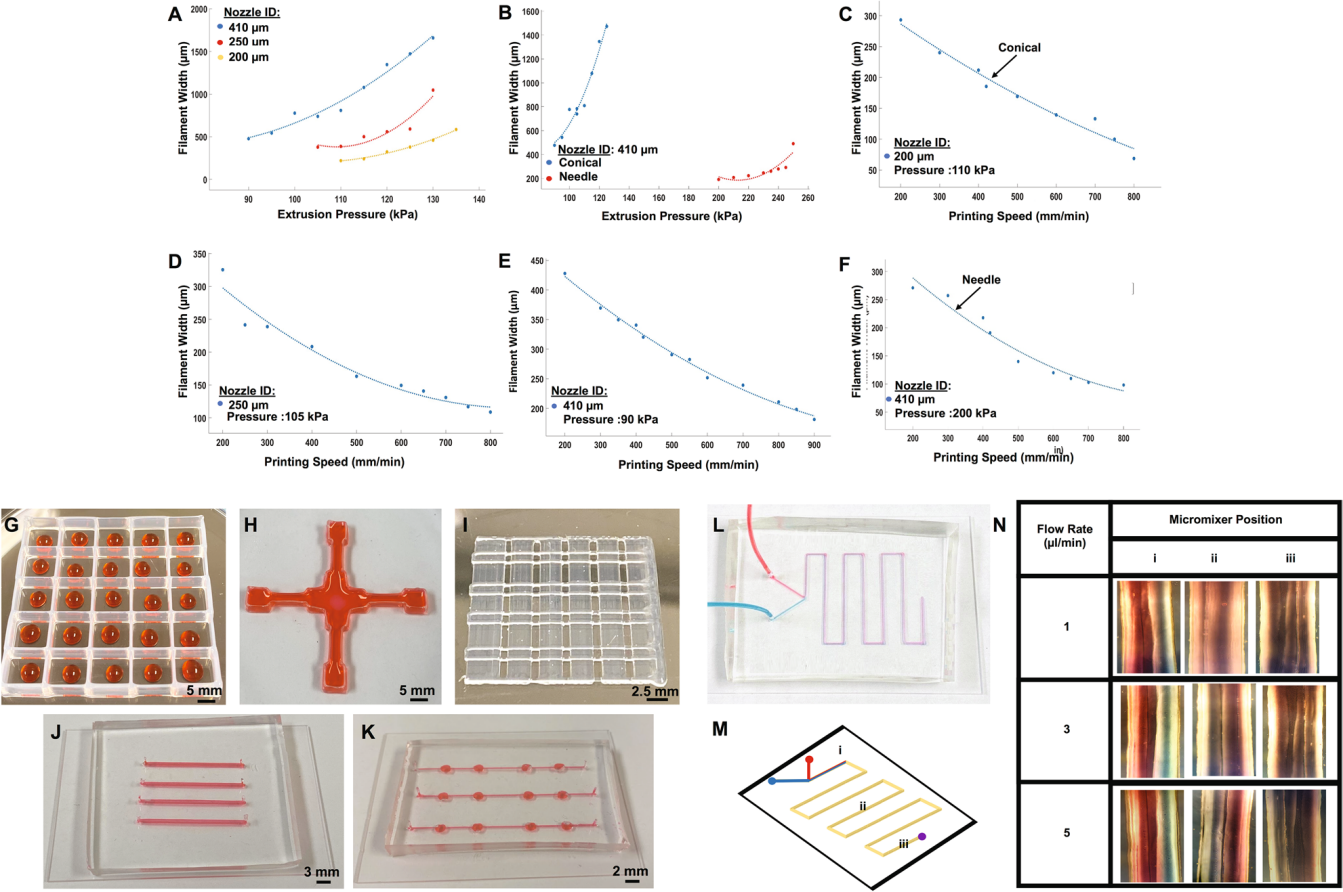
(**A**) Impact of extrusion pressure on the printed filament width for conical nozzle tips with different inner diameters (200, 250, 410 µm) (**B**) for conical and needle-shaped nozzle with same ID (410 µm). Impact of printing speed on the filament width for different conical nozzle ID: (**C**) 200 µm, (**D**) 250 µm, and (**E**) 410 µm and (**F**) needle-shape nozzle ID:410 µm. PDMS printed configurations of (**G**) 10 mm × 10 mm × 3 mm (L × W × H) well array (**H**) cross shape (**I**) and grid well structure. Images of PDMS-based (**J**) microchannels, (**K**) concave well connected by channels, and a (**L**) Y-shaped micromixer derived from printed Pluronic molds. (**M**) Diagram of the Y-shaped micromixer, showing the positions where mixing between a red and blue solution were optically verified. (**N**) Images of red and blue solutions mixing for solution flow rates of 1,3, and 5 µl/min and at positions i, ii, and iii which indicate 10 mm,75 mm and 145 mm distance from the inlet respectively.

The rapid and precise manufacturing of OOC devices remains a significant hurdle in their implementation as novel platforms for in vitro disease studies and therapeutic screening. Here, we used micro extrusion-based 3D-bioprinting and its short turnover times for the customized (i.e., different geometries and dimensions) production of fluidic handling systems, as a component of our OOC devices. To do so, we first performed characterization experiments that are required to find the optimum 3D-bioprinting parameters for Pluronic F-127. Pluronic F-127 is a useful class of synthetic block copolymers that is biocompatible and works well with PDMS^45^. For initial characterization experiments, we first determined the minimum extrusion pressures required to achieve continuous filament printing for the different nozzle diameters. According to our results, the minimum extrusion pressures required were 90, 105, and 110 kPa for conical nozzles with IDs of 410, 250, and 200 µm, respectively (Fig. 1A). Next, we evaluated the minimum extrusion pressure for different shaped nozzles with the same inner diameter (ID:410 µm) (Fig. 1B). It was found that needle-shaped nozzle required a minimum extrusion pressure of ~ 200 kPa, while the conical nozzle necessitated ~ 90 kPa. Once the minimum extrusion pressures were known for each respective nozzle ID and shape, the pressure was fixed at those values and the effect of printing speed on filament feature widths was studied for conical nozzles with ID of 200 µm (Fig. 1C), 250 µm (Fig. 1D), 410 µm (Fig. 1E), and the needle nozzle with ID of 410 µm (Fig. 1F). From this analysis, we determined that higher printing speeds, yield minimum filament widths of 70, 107, 184, 97 µm for conical nozzle with ID of 200, 250, and 410 µm, as well as, the needle nozzle with ID of 410 µm, respectively. From this data, we are able to determine optimum printing parameters for the desired minimum filament resolution of our printed structures.

#### Characterization and validation of the micromixers

In vitro drug screening systems should have the capability to examine different doses and a variety of therapeutics on the same model population. The incorporation of microfluidic devices such as micromixers allows for the preparation and delivery of different concentrations or (combinations of) therapeutics within the chip flow circulation^46^. In particular, Y-shaped passive micromixers are simple in design, rapid, and usually high performance. These features make them suitable candidates for a wide variety of ‘‘lab-on-a-chip’’ applications, where a rapid and homogenous mixing process is essential. Here, as a model of study, we designed and validated a low-cost and rapidly prototyped Y-shaped micromixer (Fig. 1L,M). The micromixer was manufactured from Pluronic molds that were printed with the dimensions of 800 ± 50 µm wide, 80 ± 10 µm height, and composed of two inlet streams and one outlet stream (Fig. 1L). The micromixer's performance was then validated at three different flow rates of 1, 3, and 5 µL/min. The inlet streams were infused with red and blue dye deionized (DI) water solutions. As shown in Fig. 1M,N, at the flow rates < 3 µl/min, the homogenous mixing of the solutions occurs at ~ 75 mm distance (position ii) from the inlet (Fig. 1M). While higher flow rates > 5 µl/min require longer distances, and the homogenous mixture was observed at approximately 145 mm (position iii) from the inlet (Fig. 1M). Also, at low flow rates < 3 µl/min, the flow of micromixer mostly is laminar, and the molecular diffusion is dominant. However, by increasing the flow rate, longer mixing time is required for interfusion of two solutions in mixing zones. The threshold flow rate of proper homogenous mixing in our device was experimentally determined to be 7 µl/min.

#### 3D-manufacturing of high throughout PDMS well arrays

**Figure 2:**
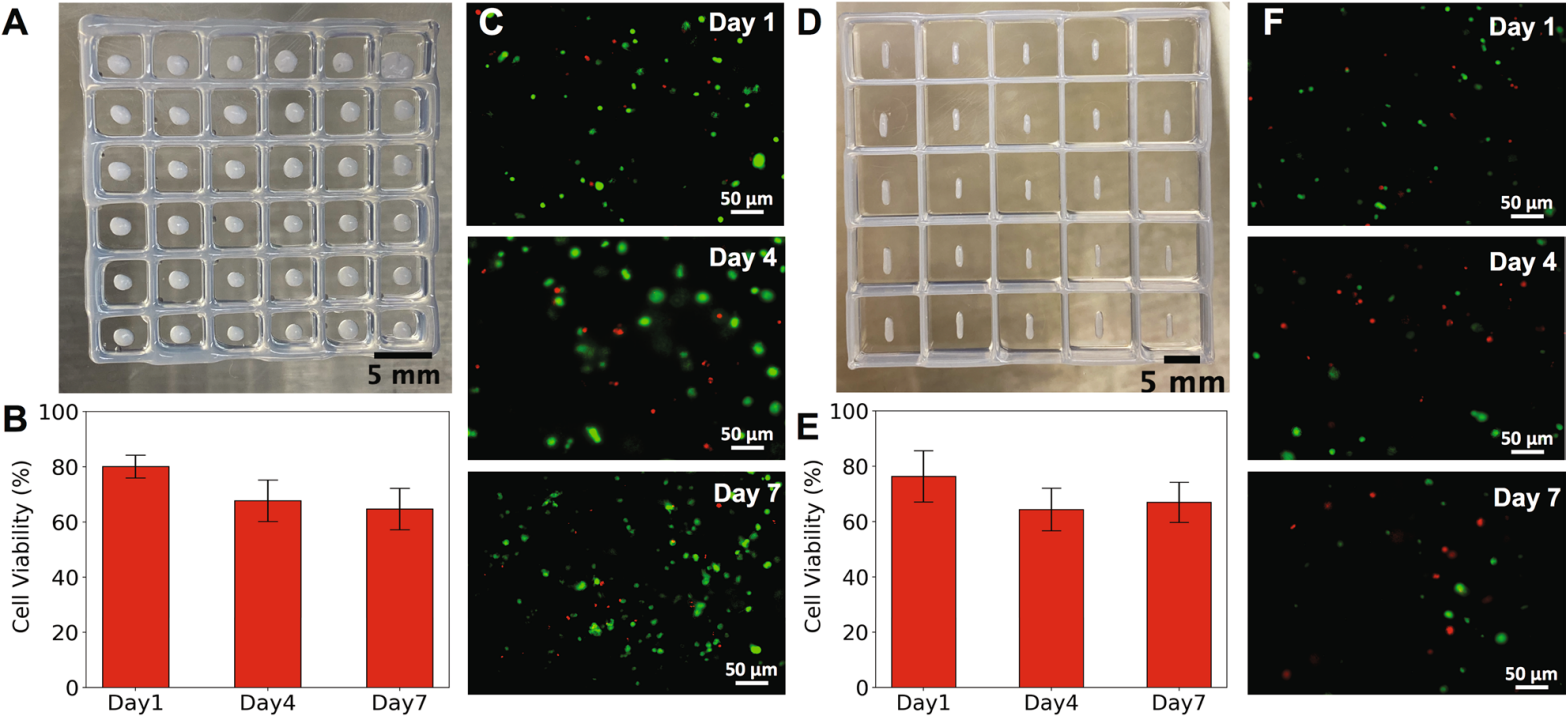
(**A**) Image of 3D printed structures composed of bioink and HCT116 in forms of spheres. (**B**) Bar plot showing the maintained cell viability within spheres geometry constructs for 7 days. The viability alters by ~ 19% of the day 1 viability. (**C**) Fluorescent image representatives of stained spherical HCT 116 cells-bioink constructs, on day 1,4, and 7, where green are live cells and red are dead cells. (**D**) Image of 3D printed rectangular structures composed of bioink and HCT116 cells. (**E**) Bar plot showing the maintained cell viability within rectangular constructs for 7 days. The viability alters by ~ 12% of the day 1 viability. (**F**) Fluorescent image representatives of rectangular stained HCT 116 cells-bioink constructs, on day 1,4, and 7.

OOCs in vitro drug testing platforms require array-based and multiplex systems in order to achieve rapid and high throughput performance. Here, we demonstrated the direct 3D-bioprinting of PDMS well-based arrays for the analysis of 3D-cell culture models. We are capable of printing a wide range of geometries of PDMS wells, as observed in Fig. 1G–J, including square wells, cross configurations for multidirectional perfusion, and small well grid structure. However, to analyze different printed 3D-cell constructs and cell populations in our studies, three different sizes of 3D-printed PDMS well arrays were designed and directly printed. The design and direct printing of the PDMS well arrays showed the adaptability of 3D-bioprinting for the integration of bioanalytical platforms. Then, 3D-spherical HCT 116 cell constructs were printed and analyzed within 5 mm × 5 mm × 3 mm (length, width, height) well arrays (Fig. 2A), rectangular HCT 116 cell 3D-constructs were printed and analyzed within 10 mm × 10 mm × 3 mm well arrays (Fig. 4D), and 2D HCT 116 models were analyzed within 15 mm × 15 mm × 3 mm 3D-printed PDMS well arrays (B), as explained in “3D-Printability of 3D-HCT116-constructs” and “Preliminary drug screening of SN-38 on 2D HCT116 cell models within 3D-PDMS bioprinted well arrays”. One should note that conventional microfluidic device production approaches usually require the design of multiple non-tailorable masks and multi-steps of photolithography to pattern and generate microstructure molds^47,48^. Those processes are then followed by PDMS casting, degassing, and curing steps to produce the final PDMS-based microfluidic device. In contrast, our process is a single step, rapid, inexpensive, and tailorable design process. Here there is no need for lithography masks or performing any complicated microfabrication techniques. CAD designs can be given to a 3D-printer to simply, rapidly, and directly print any desired configuration of the PDMS well arrays. Our approach enables rapid and cost-effective production of microfluidic devices with a variety of dimensions, > 30 µm. It makes our process an ideal process for rapid prototyping of microfluidic device integration (with a printing time that is less than 5 min).

### 3D-bioprintability and viability monitoring of 3D-HCT116-constructs

#### 3D-bioprintability of 3D-HCT116-constructs

3D-models have shown to better predict the success of drug treatments in preclinical trials, due to the improved biological microenvironment relevance compared to 2D-culture models. In this work, we demonstrated the 3D-bioprinting of 3D-construct models of HCT116 cells. HCT116 cells were selected as a model for colon cancer tumors. Despite the advancements in colon cancer treatment, therapeutic drug investigations for those patients that present drug resistance is still necessary. For the HCT116 models, two different geometries were generated, a spherical and rectangular structure (Fig. 2A,D). These models were homogeneously printed from a mixture of HCT116 cells and Cellink bionk at the ratio of 1:10. The bioink, which is composed of alginate and nanofibrillar cellulose allowed for HCT 116 cells to be in an environment that closely resembles their native ECM in the human body. The Cellink bioink also provided a representation of possible in vivo drug transport (delivery), as anti-cancer drugs often must permeate through a mixture of tissue and ECM to reach the tumor. Mixing cells with the bioink and printing structures without any trapped-air-bubbles is usually a critical issue in bioprinting. One should note that even a small amount of trapped-air-bubbles in the cells-bioink mixture may affect the bioprinting parameters. Our printing process was optimized until no bubble formation in the printed cell mixture was observed (Nozzle 410 μm, extrusion pressure 4 kPa, printing speed 100 mm/min).

#### Viability analysis of 3D-HCT116-constructs

Previous studies in 3D-bioprinting have shown there is a large range of cell survival^24^. The cell survival rates depend on the level of shear stress that cells are subjected to during extrusion^49^. Subsequently, monitoring post-printing cell viability is crucial to gauge the success of different structures printing. In this part of the work, we sought to validate and monitor the viability of bioprinted spherical and rectangular 3D-structures within bioprinted PDMS well arrays. The viability of HCT 116 cells within spherical and rectangular constructs was monitored and imaged for 1, 4, and 7 post-printing, as shown in Fig. 2. For viability assessment (Fig. 2B,E), fluorescent images for live and deal labeled cells were imaged at three z-distances and for three different structures to obtain the best results (Fig. 2). All cell viability data are presented as mean values ± standard deviation. The cell viability percentage of spherical 3D-cell constructs was measured as 80.1 ± 4.1%, 67.8 ± 7.5%, and 64.7 ± 7.5% for 1, 4, and 7 days post-printing, respectively. Similarly, the rectangular HCT 116 3D-cell constructs were examined for 1, 4, 7 days post-printing, as shown in Fig. 2E,F. The average cell viability for these constructs was measured as 76.3 ± 9.2%, 64.4 ± 7.6%, and 67 ± 7.3% for 1,4, and 7 days post-printing, respectively.

#### Inclusive fluidic handling system along with 3D-HCT116-constructs

**Figure 3:**
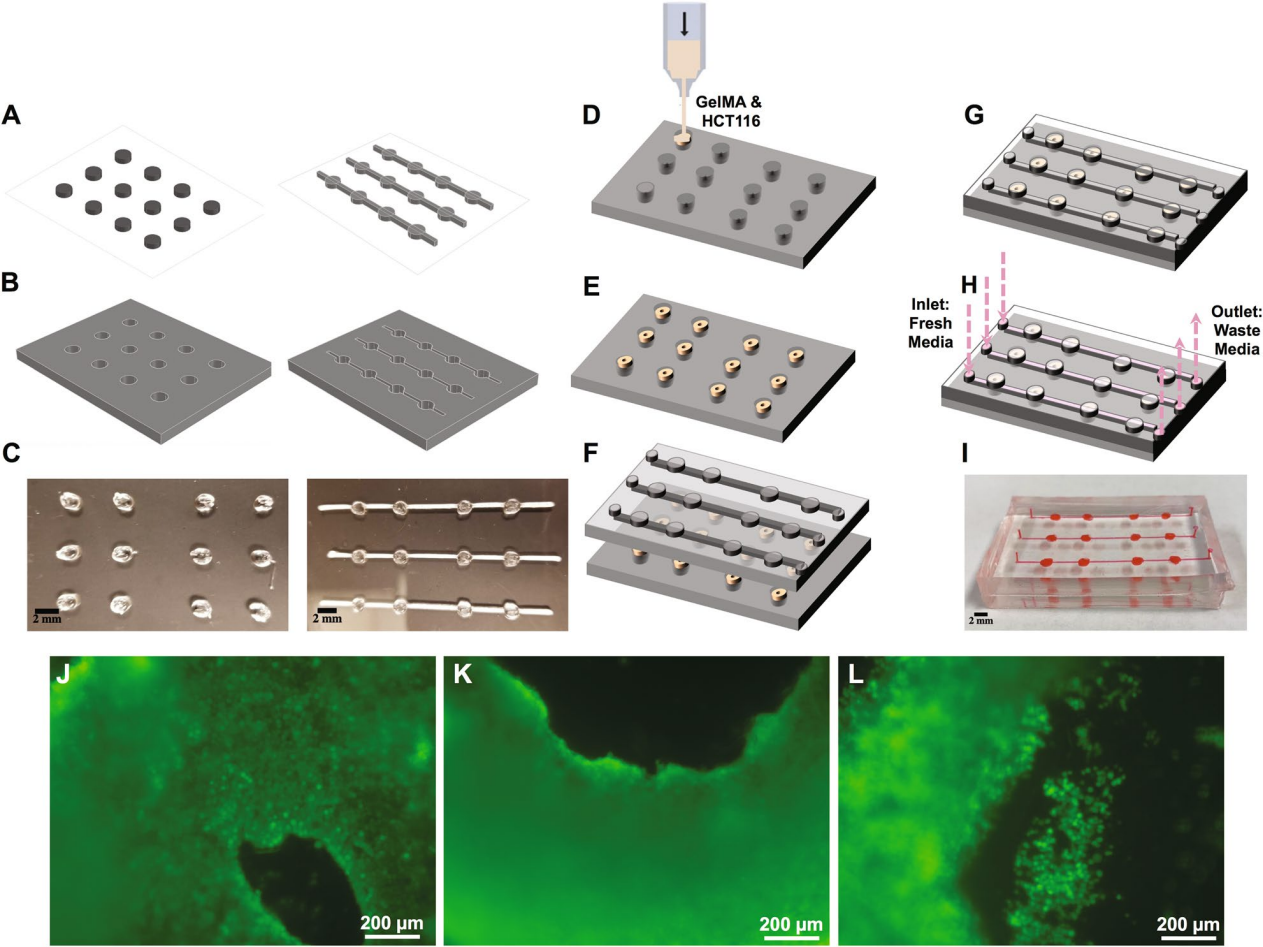
(**A**) Schematics of printed Pluronic molds and resulting (**B**) PDMS casts for concave wells and channels. (**C**) Image of printed Pluronic molds used to fabricate PDMS concave wells and channels (scale bar: 2 mm). The process of (**D–E**) 3D printing GelMA-HCT116 structures within concave wells, (**F–G**) assembling the microfluidic platform, and (**H**) media perfusion of GelMA-HCT116 structures**. **(**I**) Photograph of the concave well-based microfluidic platform (scale bar: 2 mm). (**J–L**) Image representatives of three different toroidal formed structures of the 3D-bioprinted GelMA and HCT116 cell mixture. Live HCT 116 cells within the constructs were labeled with Calcein AM. Image representatives show the toroidal GelMA and HCT116 constructs with (**J**) smaller, (**K**) larger inner cavity, and (**L**) small cell island formed within the inner cavity of the ring.

Preliminary experiments consisted of 3D-cell constructs within 3D-printed PDMS wells. An open well system allows for the simplification of construct manipulation and observation during the initial testing stage. Nevertheless, well-based systems lack the replication of mechanical forces such as fluid flow (i.e., shear stress) that tissues are subject to within the body. In these experiments, we demonstrated that we are able to take all our initial results to generate a process for the production of an inclusive OOC-like device with a 3D-HCT116 culture model using a 3D-bioprinter. This device can be described as a concave well-based microfluidic platform with connecting microchannels (Figs. 1K, 3I)**.** The composition of the microfluidic platform is made up of two parts: microfluidic channels and concave wells. The channel’s width, length, and depth are 800 µm, 30 mm, and 300 µm respectively. The concave well diameter is 1.5 mm*.* The microfluidic channels enable the application of physiological fluid flow onto cell constructs while refreshing nutrients. The concave well array was designed to hold the 3D-HCT116 cell constructs. Both the channels and wells were similarly fabricated from 3D-printed Pluronic ink molds to which PDMS was then casted onto the molds to form the final structures of channels and wells (Fig. 3A–I).

Unlike the previously presented 3D-constructs, cell structures here were 3D-bioprinted from GelMA and HCT116 cells. Once printed, toroidal structures of GelMA HCT 116 cell structures were achieved (Fig. 3J–L). These toroidal structures (especially if stacked) have the potential to model the tubular geometry of the colon. They mimic tumors that are found attached to the inner wall of the large intestine. The microchannels were then added to the well array substrate where the GelMA cell structures were perfused with media. The simplified well-based perfusion design we demonstrated here can potentially be redesigned to add more channels, valves, and features that replicate human physiology such as cell–cell interactions, or delivery of gradient growth factors.

#### Preliminary drug screening of SN-38 on 2D-HCT116 cell models within 3D-PDMS bioprinted well arrays

**Figure 4:**
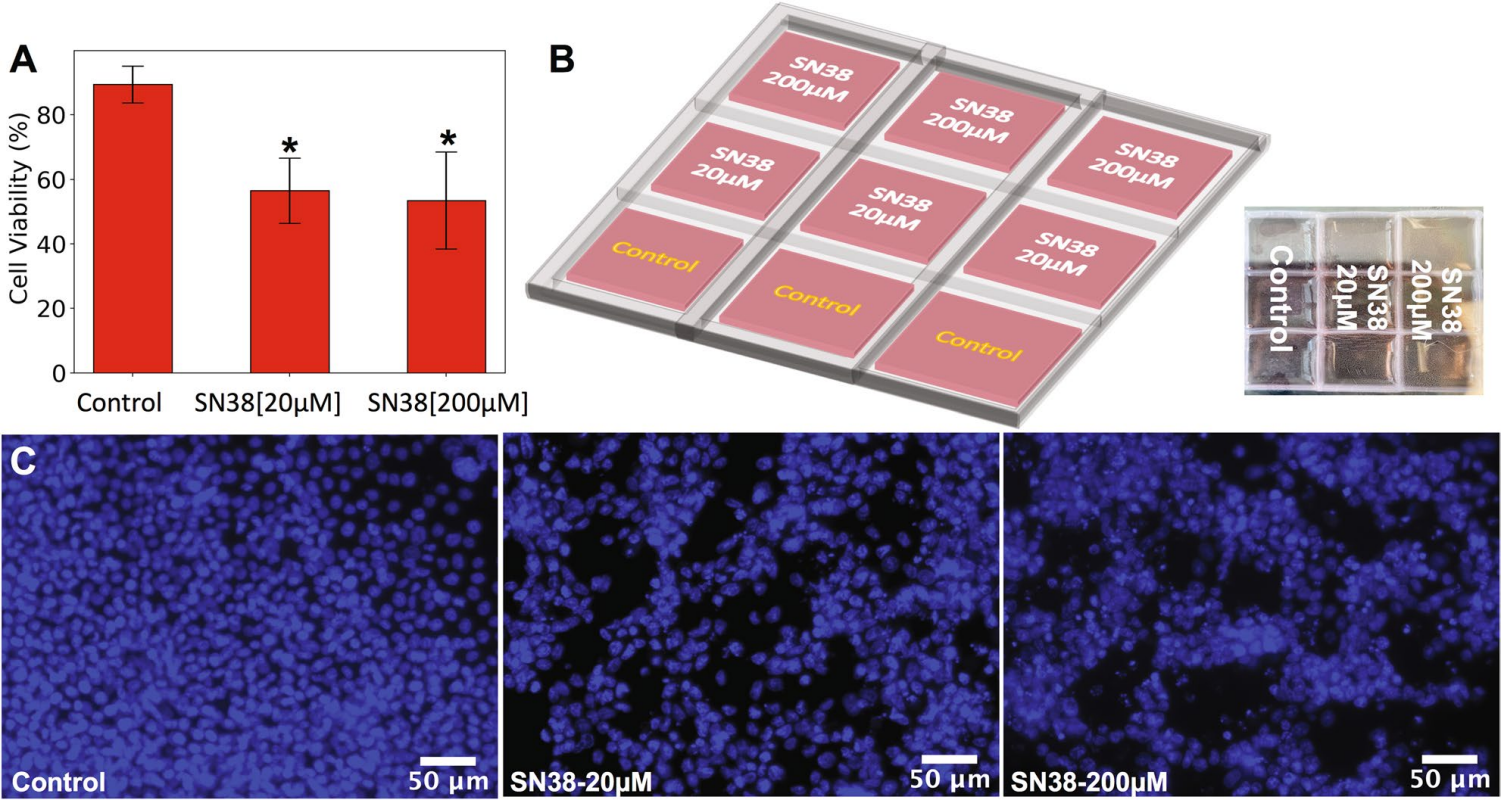
(**A**) Bar plot showing the cell viability within 2D-constructs after 48 h of drug treatment. The control cell populations showed a viability of 90%, cell populations treated with 20 µM of SN38 showed a viability of 57%, and cell populations treated with 200 µM of SN38 showed a viability of 48% after 48 h. (**B**) Schematic and image of 3D-printed PDMS well arrays with HCT 116 cultured cells. A total of three rows of wells were used, where one row (consisting of three replicate wells) was designated as a control population (no drug), a second row consisted of HCT116 cell population treated with 20 µM of SN38, and a third row consisted of HCT116 cell population treated with 200 µM of SN38. (**C**) Fluorescent image representatives of NucBlue stained HCT116 cells for control population (left), HCT116 cells with 20 µM of SN38 (middle), and HCT116 cells with 200 µM of SN38 (right) are shown, where control population shows a more cohesive adhered monolayer, while those treated with SN-38 presented significant loss of monolayer distribution with large sparse gaps. One-way ANOVA indicated that statistical significance of *p < 0.0001 for both treated populations (20 and 200 µM of SN38) compared to control population.

3D- PDMS printed well arrays were used to execute initial drug toxicity studies of 7-Ethyl-10-hydroxycamptothecin (SN-38) on 2D-HCT116 cell models. SN-38 is a drug used for colon cancer, which has the effect of an apoptotic inducer, topoisomerase I inhibitor. In this work, we used the PDMS well arrays to treat an array of HCT 116 cell populations to two concentrations of 20 µM and 200 µM of SN38 as well as maintain an array of control cell populations (Fig. 4B). Cell viability measurements after 48 h of drug treatment indicated that control cell populations have the viability of 90%, while cell populations treated with 20 µM of SN38 have a viability of 57%, and those treated with 200 µM of SN38 have a viability of 48% (Fig. 4A). Figure 4C shows the image representatives of fluorescently labeled HCT116 cell, and it observed that the control population remains adhered to the surface while cells treated with increasing SN38 concentration detach from the surface, leaving behind a less dense cell population. For the data presented here 3 different measurements were taken and are presented as mean values ± standard deviation. The one-way analysis of variance (ANOVA) determined statistically significant differences between the means of control’s cell viability and the addition of drugs with different concentration (20 μM and 200 μM), where statistical significance was shown as *p < 0.0001 for both treated populations.

## Conclusion

Expensive and failed drug clinical trials that emerge from successful animal and 2D-cell studies have driven the need for more physiologically relevant, and low-cost drug screening approaches. In this work, we have demonstrated the generation of new drug testing platforms using 3D-bioprinting technology to produce both (1) cell models that more closly mimic the microenvironment of cells and (2) flexibly and easily prototyped cell handling structures. We report the implementation of 3D-bioprinting technology to print comprehensive analysis platforms. Our experiments consisted of printing 3D rectangular and spherical cell constructs within 3D-printed PDMS well arrays. We performed a post-viability analysis, which showed that 3D-cell constructs differ in viability from 12 to 19% compare to day one. However, well-based systems lack the replication of the mechanical cues such as fluid flow (i.e., shear stress) that tissues are subject to within the body. To mimic that environment, we showed that we are able to take all our initial experiments to streamline the process of the generation of an inclusive organ-on-a-chip (OOC)-like device with the generation of 3D-GelMA HCT116 culture model using our 3D-bioprinter. Our device is a perfused well-based microfluidic platform, formed by microfluidic channels and concave wells. The microfluidic channels were designed to subject cell constructs to physiological fluid flow and, at the same time, deliver nutrients. GelMA HCT 116 cell structures were printed into the wells and formed ring/toroidal shapes within a day after printing. These tube-like structures (especially if stacked) are useful as they have the potential to model the similar geometry of the colon. Although we showed a well-based perfusion design here, it can potentially be more intricately designed and combined with the shown micromixer to induce more representative of human physiology such as cell–cell interactions and delivery of gradient growth factors or more complicated fluid-flow schematics for the delivery of different drug rates and concentrations. Finally, to demonstrate an initial drug study within our 3D-printed PDMS-based well arrays, we showed the effects of SN38 at two different drug concentrations (20 µM and 200 µM) on a 2D-cell HCT 116 cell model. This study showed that the treatment of 48 h SN-38 decreases cell viability to 48% compared to 90% cell viability observed in the control cell population.

## Methods

### Ink preparation and bioprintability analysis

The Inkredible + 3D-bioprinter (Cellink, Sweden) was used in our studies. Pluronic F127(Cellink, Sweden) was used as the printing material to create the molds used for casting the PDMS structures^50^. Pluronic is a printable ink with a wide range of applications such as low molecular weight component fabrication to sacrificial material or support structures, useful to create microchannels and vascularized tissues^51^. Pluronic does not adhere to PDMS, which makes it a suitable candidate to be used for the microchannel master molds fabrication^39^. To print molds, the bioprinter cartridge was carefully filled with Pluronic to avoid introducing any air bubbles. To find the optimum printing conditions, four nozzle tips with different inner diameters (ID) were used: a needle-shaped stainless steel nozzle (ID: 410 μm), a conical nozzle (ID: 410 μm), a conical nozzle (ID: 250 & 200 μm). To print, the bioprinter was connected to an air compressor that controls and regulates the extrusion pressure of both printheads. The patterns were designed with CAD software and exported as stereolithography (STL) files and converted to G-code with the Cellink Heartware (Inkredible + 3D-printer operating software). To validate the utility of our method, different combinations of nozzle tips, extrusion pressure, and printing speed were tested. For each combination, seven lines were printed on a petri dish surface, and the width of each line was measured. The average of the seven measurements defined the filament width.

### Ink characteristics

The used bioinks in this study were Pluronic F-127, Cellink bioink, and gelatin methacryloyl (GelMA). Pluronic F-127 is a class of synthetic block copolymers which consist of hydrophilic polyethylene oxide (PEO) and hydrophobic polypropylene oxide (PPO) copolymer^45^, Cellink bioink is composed of alginate and highly hydrated cellulose nanofibrils with morphological similarity to collagen, which mimics the milieu of the native ECM of mammalian cells^52^, and GelMA is a semi-synthetic hydrogel that consists of gelatin derivatized with methacrylamide and methacrylate group, and it can maintain shape and stability at physiological temperature and mimic an optimal microenvironment in comparison to other ECM materials^53^.

### 3D-printing of mixer modules

A laminar Y-shaped micromixer was studied. The micromixer mold was printed using Pluronic F127, a proper nozzle at 100 kPa extrusion pressure. To fabricate the PDMS micromixer, Sylgard 184 base and curing agent (Dow Corning, Auburn, MI, USA) were mixed in 10:1 proportion. The mixture was degassed and poured on the printed mixer molds. The PDMS channels were then cured in an oven at 40 °C for 12 h. It was followed by peeling off the PDMS channels from the molds. Inlet and outlet holes were punched through the channels using biopsy puncher. The PDMS channels were then cleaned by sonicating them in Isopropyl alcohol (IPA) for 5 min. To improve glass-PDMS binding, the PDMS surface was first coated with an undiluted amine-PDMS linker at room temperature for 1 h. Then the channels were sonicated in IPA to remove the excess amine-PDMS. Channels were dried using compressed air. Next, both PDMS channels and the glass substrates were activated using plasma wand discharge for 1 min, then was pressed together and placed in an oven at 75 °C to complete the bonding process. The micromixer system was completed by insertion of silicone tubing (Microbore PTFE Tubing, 0.022"ID × 0.042"OD) into the PDMS channels’ inlet and outlet holes. To characterize the mixers, blue and red-dyed (Gel food colors, Wilton, USA) were mixed with deionized water and were injected into the micromixer at different flow rates using a syringe pump (Harvard Apparatus, Pump 11 Pico Plus Elite, MA, USA).

### Fabrication of PDMS well arrays

PDMS was prepared by blending two silicone elastomers, including a low-viscosity PDMS material Sylgard 184 and a shear-thinning PDMS material SE 1,700 (Dow Corning, Auburn, MI, USA) which is used to dilute Sylgard 184 for desired rheological properties. Both SE 1,700 and Sylgard 184 base materials were mixed with their curing agents for at least 10 min in a 10:1 (base: curing agent) ratio by weight before blending and were placed in a vacuum desiccator for degassing for 15–20 min just after mixing the base and agent. Then, SE 1,700 and Sylgard 184 were mixed in an optimized ratio and placed in a vacuum desiccator for 10 min. Next, the PDMS mixer was loaded into a 3-cc syringe (barrels syringe, Cellink) at room temperature and centrifuged at 5,000 rpm for 5 min to remove any air bubbles. The proper nozzle at 140 kPa extrusion pressure was used to print the PDMS wells. To be adaptive with different sizes and shapes of the bioprinted structure, three different constructs of PDMS well array were printed. The wells with 5 mm x 5 mm x 3 mm (Length, width, height) dimensions were printed for sphere bioprinted structures, and 10 mm x 10 mm x 3 mm wells were fabricated for printing rectangular bioprinted structures. Also, 15 mm x15mm x 3 mm wells were printed for 2D-drug screening experiments. Once printed, the PDMS well arrays were cured in an oven at 75 °C for 4 h.

### Fabrication of concave well-based microfluidic platform with connecting microchannels

Concave PDMS well arrays and microfluidic channels molds were designed using AutoCAD software and converted to G-code for printing. A proper nozzle at 100 kPa extrusion pressure was used to print the Pluronic molds for both concave shape arrays and microchannels. To prepare the PDMS for casting on molds, Dow Corning Sylgard 184 base and curing agent were mixed in 10:1 proportion. The mixture was degassed in a desiccator connected to the vacuum for 15 min and subsequently poured onto the Pluronic molds. The PDMS channels were then cured in an oven at an optimized temperature for 12 h. It was followed by peeling off the PDMS channels from the molds. Inlet and outlet holes were punched to allow fluid access through the microchannel. The microfluidic structures were then sanitized using (IPA) for 30 min under the culturing hood and prepared for cell printing.

### Cell preparation

Human colon cancer cells (HCT116 cells) were used as a model of study. HCT116 cells were grown in Dulbecco's Modified Eagle Medium (DMEM, Gibco, Life Technologies, USA), Fetal Bovine Serum (FBS, Sigma-Aldrich, USA) and Antibiotic–Antimycotic (Anti-Anti, ThermoFisher Scientific, USA) and were cultured in tissue culture flasks and maintained at an incubator. Confluent flasks were washed with Phosphate-Buffered Saline (PBS) (Gibco, Life Technologies, USA) and trypsinized to harvest cells for each experiment.

### 3D-bioprinted sphere and rectangular structures inside the PDMS well array

Cellink bioink (Alginate and Nanofibrillar cellulose bioink) was used for 3D-bioprinting experiments. The printing procedure is as follows: first, the Cellink bioink (3 ml) was loaded into a 3 ml syringe, and cell suspension (429 μl) was loaded into a 1 ml syringe (7:1 mixing ratio) using a female/female luer lock adaptor. Both syringes were then connected to each other using female/female luer lock adaptor.The bioink and the cell suspension were mixed by gently pushing the bioink and cells back and forth between the syringes then were dispensed on a 3 cc cartridge and created a homogeneous distribution of cells in bioink by applying gentle pressure. Once the cartridge was filled, the nozzle was connected to it, and the cartridge was placed in the printhead. Then the mixture of bioink and cells were printed in PDMS wells in two different shapes, sphere structure in 5 mm × 5 mm × 3 mm PDMS well array, and rectangular structures were printed in 10 mm × 10 mm × 3 mm. After printing, the cell-laden structures were cross-linked with ions using the 200 µl of CaCl_2_ crosslinking solution (100 mM). Next, cell-laden structures were rinsed with PBS, and cell culture media were added. Then, the printed structures were placed in an incubator, and culture media was changed every other day.

### Bioprinting GelMA + cell in PDMS concave well array

In another platform, GelMA was mixed with cells and was printed inside the PDMS wells array, and the PDMS microfluidic channel that allows for media perfusion was bounded on top of it. The printing procedure is as follows: first, the GelMA in the cartridge was heated up to 37 °C in the incubator until it was liquid. It was tested, flipping the cartridge and observing if air bubbles move freely. Then, the GelMA (1 ml) was loaded like bioink into a syringe. The process of mixing and printing should be performed rapidly before GelMA becomes too viscose. The temperature of the printhead was set to 26 °C. Once the cartridge was filled, the nozzle was connected to it, and the cartridge was placed in the printhead. The minimum extrusion pressure (2 kPa) was selected to have continuous printing filaments and minimizing shear stress. The mixture of GelMA and cells were printed in PDMS concave shape and was cross-linked using a 405 nm photocuring module (UV). Next, cell culture media were added to the bioprinted structure and were placed in an incubator. The day after printing, the microchannels was treated using a corona discharge wand and was bounded on top of the PDMS well array. Afterward, the media was dropped on the channels’ inlet and outlet, and the channels were filled with media by sucking out from the channel’s outlet.

### Drug screening of 2D-HCT116 cell models

For 2D-culture, the PDMS well arrays (15 mm × 15 mm × 3 mm) were used. The culturing surface was coated with Fibronectin (FN1, Sigma Aldrich, USA), which plays an important role in cell attachment and spreading, control of morphology, and differentiation. The fibronectin was diluted to 20 μg/mL in PBS and placed into well for 45 min at room temperature. Then HCT116 cells were seeded at an approximate concentration of 400,000 cells in each well with 500 μL medium and were placed in an incubator overnight. They were then rinsed with PBS to remove any unadhered cells. Two different concentrations of SN-38 (20 μM and 200 μM) were prepped in DMEM and added to the wells for 48 h. For the control cell population, only media was added.

### Cell viability and imaging

Bioprinted colon cancer cells (HCT 116) viability, inside a sphere and rectangular bioprinted structures, were monitored after 1, 4, and 7 days. Propidium Iodide (PI) dye (Invitrogen, Thermo fisher scientific, USA) was used as a dead cell indicator and Calcein AM fluorescence dye (eBioscience™ Calcein AM Viability Dye, UltraPure Grade, Thermo fisher scientific, USA) was used as a live cell indicator. The number of alive and dead cells over time was monitored using a fluorescent microscope. The structures’ pre-imaging preparation and imaging process were as follows: first, the cell-laden structures were removed from the incubator, the existing media was aspirated, and the structure was washed off with fresh Hank's balanced salt solution (HBSS). Next, 2 µM Calcein AM dilution in DMEM, which stains the live cells, was added to cell-laden and, after incubating for approximately 40 min, was aspirated. Next, Propidium Iodide, which stains the nuclei of dead cells were mixed with PBS (2 drops/1 ml PBS) to obtain dead cell imaging solutions. The solutions were added to the cell-laden structure and incubated for another ~ 20 min. Finally, the structures were washed off using HBSS twice (each time 15 min) before the imaging process started. Five random individual wells were selected and used for the imaging analysis. It was followed by analyzing the live and dead cells by using ImageJ software. The images were then carefully thresholded to highlight the region of interest and then analyzed for the number of dead and live cells. The staining protocol and imaging process for the GelMA structures within microchannels were the same, just for the staining, first, the media was sucked up from the outlet, and the stain solution was injected into the channels from the inlet. For 2D-HCT116 cell models, NucBlue (Invitrogen, Thermo fisher scientific, USA) reagent, which stains all of the cells and propidium iodide (dead cell indicator), was added directly to cells in media (both and placed in the incubator for 20 min. Before starting imaging, the media was aspirated from each well. Images were captured of different spots of wells. The images were analyzed using ImageJ software.

### Statistics

All data are presented as the mean ± standard deviation (SD). Drug screening of 2D-HCT116 cell model’s data were analyzed by GraphPad prism using one-way analysis of variance (ANOVA) to test for significance when comparing the control’s cell viability and the addition of drugs with different concentration. Posthoc Tukey’s multiple comparison test was used to determine the individual differences among the groups. Statistical differences were considered at p < 0.0001 (*).
